# Dose‐Optimization of a Novel Co‐Formulated Triple Combination Antimalarial Therapy: Artemether‐Lumefantrine‐Amodiaquine

**DOI:** 10.1002/cpt.3582

**Published:** 2025-02-12

**Authors:** Joel Tarning, Nicholas J. White, Arjen M. Dondorp

**Affiliations:** ^1^ Mahidol Oxford Tropical Medicine Research Unit, Faculty of Tropical Medicine Mahidol University Bangkok Thailand; ^2^ Centre for Tropical Medicine and Global Health, Nuffield Department of Medicine University of Oxford Oxford UK

## Abstract

Artemisinin‐based combination therapy (ACT) is the first‐line therapy for uncomplicated *falciparum* malaria, but artemisinin resistance in Asia and now sub‐Saharan Africa is threatening our ability to control and eliminate malaria. Triple‐ACTs have emerged as a viable alternative treatment to combat declining ACT efficacy due to drug‐resistant malaria. In this study, we developed and evaluated an optimal fixed‐dose regimen of artemether‐lumefantrine‐amodiaquine through population pharmacokinetic modeling and simulation. Three published population‐based pharmacometric models and two large cohorts of observed adult subjects and pediatric malaria patients were used to simulate pharmacokinetic profiles of different dosing strategies. Based on simulated total exposure and peak concentrations, an optimal dose regimen was developed resulting in an extension of the current 4 weight bands to a total of 5 weight bands to generate equivalent exposures in all body weight groups and minimize the fluctuation in exposure between patients. The proposed drug‐to‐drug ratio of artemether‐lumefantrine‐amodiaquine (20:120:40 mg) was kept constant throughout the dosing bands in order to simplify manufacturing, implementation, and further development of a fixed‐dose co‐formulated product.

**Study Highlights**

**WHAT IS THE CURRENT KNOWLEDGE ON THE TOPIC?**

Artemisinin‐based combination therapy (ACT) is the first‐line therapy for uncomplicated *P. falciparum* malaria. Artemisinin‐resistant *P. falciparum* was first identified in Southeast Asia, but artemisinin resistance has recently emerged in East Africa. Triple‐ACTs have emerged as a viable alternative treatment to combat declining ACT efficacy due to multi‐drug‐resistant malaria.

**WHAT QUESTION DID THIS STUDY ADDRESS?**

Artemether‐lumefantrine is the most commonly used antimalarial today and has been proposed for development into a fixed‐dose combination with amodiaquine to address potential adherence issues associated with loose‐tablet regimens. This study aimed to select and evaluate a novel fixed‐dose regimen of artemether‐lumefantrine‐amodiaquine through population pharmacokinetic modeling and simulation.

**WHAT DOES THIS STUDY ADD TO OUR KNOWLEDGE?**

Based on simulated total exposure and peak concentrations, an optimal dose regimen was developed resulting in an extension of the current 4 weight bands to a total of 5 weight bands to generate equivalent exposures in all body weight groups and minimize the fluctuation in exposure between patients.

**HOW MIGHT THIS CHANGE CLINICAL PHARMACOLOGY OR TRANSLATIONAL SCIENCE?**

Using a modeling and simulation approach, we propose a fixed‐dose formulation of artemether‐lumefantrine‐amodiaquine (20:120:40 mg) to simplify manufacturing, implementation, and further development of a co‐formulated product. The proposed dosing should maximize the chance of patient cure by providing an increased artemether‐lumefantrine dose in small children and large adults while minimizing the risk of increased adverse events linked to higher doses of amodiaquine by splitting the standard once‐daily dose into twice‐daily dosing to be co‐formulated with artemether‐lumefantrine.

There were an estimated 249 million cases of malaria worldwide, leading to 608,000 deaths, in 2022. Children under the age of five accounted for 76% of the total malaria‐related deaths.^1^ Artemisinin‐based combination therapy (ACT) is the first‐line therapy for uncomplicated *falciparum* malaria, recommended by the World Health Organization (WHO). The artemisinin drug has a very potent and rapid parasite‐killing effect, resulting in a substantial reduction in the parasite biomass during the first 3 days of treatment. The long‐lasting partner drug is responsible for the elimination of residual parasites, in order to prevent recrudescent malaria infections.

Artemisinin‐resistant *falciparum* malaria was first characterized in Southeast Asia and has spread widely throughout the region in the past 15 years.^2^, ^3^ ACT partner drug resistance has now also emerged on the backbone of artemisinin‐resistant infections, resulting in poor efficacy and treatment failures of certain ACTs in Southeast Asia.^4^, ^5^ Recently, artemisinin resistance in *P. falciparum* has emerged independently in several countries in sub‐Saharan Africa.^6^ Widespread artemisinin resistance and the development of multi‐drug resistant malaria in Africa would have a devastating impact on our ability to treat and eliminate malaria.

Triple‐ACTs (TACTs) have emerged as a viable alternative treatment regimen to combat declining efficacy due to multi‐drug‐resistant malaria. Recent randomized controlled trials have shown excellent safety and efficacy of two TACTs; artemether‐lumefantrine‐amodiaquine and dihydroartemisinin‐piperaquine‐mefloquine.^7^, ^8^ Artemether‐lumefantrine is the most commonly used antimalarial today and was proposed for development into a fixed‐dose combination formulation with amodiaquine to address potential adherence issues associated with loose‐tablet regimens. Several lines of evidence suggest that lumefantrine and amodiaquine have counter‐acting mechanisms of antimalarial drug resistance, making artemether‐lumefantrine‐amodiaquine an attractive drug combination to deploy. In this study, we aimed to select and evaluate an optimal weight‐based dosing regimen of a novel fixed‐dose combination formulation of artemether‐lumefantrine‐amodiaquine through population pharmacokinetic modeling and simulation.

## MATERIALS AND METHODS

Artemether‐lumefantrine and artesunate‐amodiaquine, the two most widely recommended ACTs, are currently dosed according to body weight in four bodyweight bands (**Table** **S1** **and** **S2**). Artemether‐lumefantrine is available as a dispersible formulation or as standard fixed‐dose tablets. The World Health Organization dose range target is 5–24 mg/kg/day of artemether and 29–144 mg/kg/day of lumefantrine, given twice daily for 3 days.^9^ Artesunate‐amodiaquine is available as a standard fixed‐dose tablet formulation. The World Health Organization dose range target is 2–10 mg/kg/day of artesunate and 7.5–15 mg/kg/day of amodiaquine, given once a day for 3 days.^9^ Although used for decades with great success, these two ACTs have been shown to have reduced efficacy in children^10^, ^11^ and it has been proposed to revise and expand the four weight bands currently used to achieve a more equivalent exposure in all patient groups.^12^, ^13^

Three previously published population pharmacokinetic models were used to simulate the proposed optimal dosing regimen of the artemether‐lumefantrine‐amodiaquine TACT, compared to standard dosing.^12^, ^13^, ^14^ All three pharmacometric models were well‐characterized and based on observed drug measurements in patients with uncomplicated *falciparum* malaria. Artemether and its active metabolite dihydroartemisinin were described by a simultaneous pharmacokinetic model, assuming complete *in‐vivo* conversion of artemether into dihydroartemisinin. Both artemether and dihydroartemisinin were characterized by one‐compartment disposition models, combined with a transit‐absorption model of artemether. Lumefantrine was described by a two‐compartment disposition model with first‐order absorption. Amodiaquine and its active metabolite desethylamodiaquine were described by a simultaneous pharmacokinetic model, assuming complete *in‐vivo* conversion of amodiaquine into desethylamodiaquine. Amodiaquine and desethylamodiaquine were characterized by two‐ and three‐compartment disposition models, respectively, combined with a transit‐absorption model of amodiaquine. Disease status was incorporated as a covariate in the pharmacometric model of lumefantrine and amodiaquine, and this was included by simulating only patients with acute illness. Both the artemether and lumefantrine models included pregnancy as a covariate, which was omitted in order to simulate non‐pregnant patients in this study. The pharmacometric model describing amodiaquine and desethylamodiaquine took metabolic enzyme maturation (i.e. ontogeny) into account, which was incorporated in the simulations by patient age. Only the lumefantrine model showed dose‐limited absorption, which was incorporated in all simulations according to the individual mg/kg dose administered. All models had body weight incorporated as an allometric function to scale drug exposure according to body size differences between pediatric and adult patients. Between‐patient variability was incorporated as exponential functions on pharmacokinetic parameters, as per the original publications.

Two publicly available databases were downloaded and used in this simulation study; the National Health and Nutritional Examination Survey (NHANES III) from the US CDC (*n* = 53,833), including healthy adults and children,^15^ and the Severe Malaria African Children (SMAC) network (*n* = 26,051), including African children with severe malaria infections.^16^, ^17^ A locally‐weighted scatterplot smoothing (10‐point smoothing window) was applied to all age‐for‐weight data (**Figure** **S1**), using GraphPad Prism v.10.2.2. The resulting smoothing was used to derive an average age in each body weight group (1 kg increment between 5 and 100 kg) in order to generate virtual patients with biologically plausible weight‐age combinations. The published pharmacometric model for each drug was applied to simulate a total of 1000 patients for each body weight and dosing regimen (i.e. a total of 96,000 patients for each drug and regimen). All pharmacometric simulations were performed in NONMEM v 7.5. Simulated day 7 drug concentration, total exposure (area under the concentration‐time profile), and peak drug concentration (maximum concentration) were extracted from the simulations and summarized as medians and interquartile ranges (25^th^–75^th^ percentile), using R v.4.3.3. Clear pharmacokinetic targets are not generally available for antimalarial drugs, and dose optimization was therefore realized by aiming for an equivalent drug exposure to artemether‐lumefantrine‐amodiaquine in children compared to adults. Safety target exposures have not been established for these drugs and the simulated 75^th^ percentile of a typical adult patient receiving standard ACT dosing was used as a safety threshold. A typical adult malaria patient was assigned a body weight of 60 kg as this is commonly reported in Southeast Asian patients^2^, ^18^ and it is also the midrange of the highest weight band of the current dose recommendation of artemether‐lumefantrine and artesunate‐amodiaquine.

### Ethics statement

No ethical approval was necessary as published pharmacometric models were used in combination with publicly available databases for demographic values.

## RESULTS

To minimize the extent of modifications needed to combine two existing treatments, the current dosing ratio of artemether‐lumefantrine (20:120 mg) was retained, with the addition of 40 mg amodiaquine (**Table** **1**). The amodiaquine dose strength was chosen to generate a similar mg/kg/day dose of amodiaquine as seen with standard dosing of artesunate‐amodiaquine in a typical adult patient. This drug‐to‐drug ratio (20:120:40 mg) was kept constant throughout the dosing bands in order to simplify manufacturing, implementation, and further development of a fixed‐dose co‐formulated product. The standard 4 dosing bands were transformed into 5 dosing bands to achieve more equivalent exposure in all weight groups and to reduce fluctuations in peak concentrations. Furthermore, the proposed optimal weight‐based dosing was constructed to achieve equivalent drug exposure to artemether‐lumefantrine‐amodiaquine in small children compared to adults, while safeguarding that no patients received higher peak concentrations of amodiaquine compared to current dose recommendations.

**Table 1:** Proposed optimal dosing of the novel fixed‐dose co‐formulation of artemether‐lumefantrine‐amodiaquine

Body weight (kg)	Artemether (mg)	Lumefantrine (mg)	Amodiaquine (mg)
5 to 9.9	20	120	40
10 to 15.9	40	240	80
16 to 29.9	60	360	120
30 to 54.9	100	600	200
≥ 55	120	720	240

All doses contain a fixed ratio of artemether‐lumefantrine‐amodiaquine (20:120:40 mg). Amodiaquine dosing is given as base equivalent. Doses should be administered at 0, 8, 24, 36, 48, and 60 hours, together with food.

**Figure 1:**
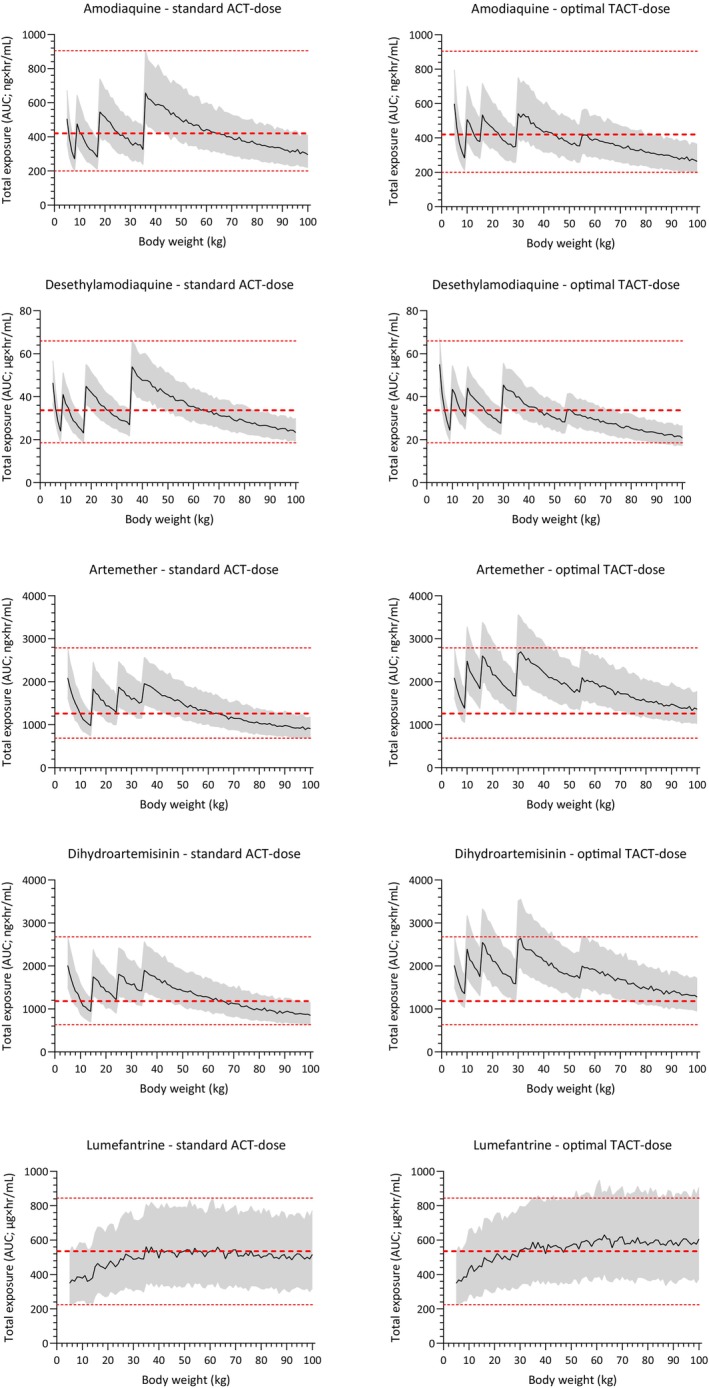
Simulated total exposure to amodiaquine/desethylamodiaquine, artemether/dihydroartemisinin, and lumefantrine after standard ACT dosing and proposed novel TACT dosing. Total exposure is derived as the area under the plasma concentration‐time profile (AUC) for each simulated individual. Each dosing simulation is based on 96,000 virtual patients, distributed uniformly between 5 and 100 kg body weight, and shown as the median (solid line) and interquartile range (gray shaded area) of simulated exposures. Dashed red lines are the median exposures in a typical patient at 60 kg, receiving standard dosing. Dotted red lines are simulated minimum and maximum interquartile exposures associated with standard dosing.

The databases used provided a large virtual population of a total of 79,884 individuals, resulting in reliable estimates of the average age‐weight relationship. The published pharmacometric models were successfully coded and a total of 1000 patients were simulated for each body weight and dosing regimen. Pharmacometric simulations showed that the proposed optimal dosing resulted in equivalent drug exposures to artemether‐lumefantrine‐amodiaquine in all weight bands (**Figure** **1** and **Figure** **S2**). Simulations also showed a small increase in total exposures and peak concentrations of artemether‐lumefantrine compared to standard dosing. Total simulated exposure to amodiaquine and desethylamodiaquine was comparable to that of standard dosing, while peak concentrations were substantially lower due to the twice‐daily dosing compared to once daily dosing in the standard dosing regimen. Simulated day 7 concentrations are often used as a proxy for total drug exposure in antimalarial drug research, as patients often return for a follow‐up visit 1 week after treatment and, therefore, allow a blood draw at this time. A total of 92% vs. 90% of patients had simulated lumefantrine day 7 concentrations above a previously reported threshold of 175 ng/mL associated with therapeutic success^19^ when receiving the proposed dosing vs. standard dosing (**Figure** **S3**). No robust threshold has been reported for desethylamodiaquine, but simulated day 7 desethylamodiaquine concentrations (**Figure** **S3**) were similar when receiving the proposed dosing (median [90% CI]: 53.5 [29.0–100.3] ng/mL) and standard dosing (median [90% CI]: 55.3 [29.9–105.7] ng/mL), and also similar to that reported in previous studies.^12^

## DISCUSSION

We used a pharmacometric modeling and simulation approach, based on previously published and validated population‐based pharmacokinetic models to derive an optimal body weight‐based dosing regimen for the novel co‐formulated triple combination therapy; artemether‐lumefantrine‐amodiaquine. All covariates described in the individual models were kept identical in all simulations of novel and current dosing scenarios and would, therefore, not influence the relative difference between dosing regimens.

The proposed dosing regimen of the triple combination therapy showed equivalent drug exposures to artemether‐lumefantrine‐amodiaquine for all body weights evaluated. This regimen also has the advantage of a somewhat higher dosing of artemether (3.0–8.0 mg/kg/day, **Table** **S3**) compared to current ACT standard dosing (2.0–5.0 mg/kg/day, **Table** **S1**). This results in a minimum dose of 4.0 mg/kg/day in children < 30 kg, who are the main patient group receiving antimalarial therapy. The minimum dose in this group of pediatric patients is currently 2.2 mg/kg/day when following the standard ACT dosing recommendation, which is substantially lower than the target dose of 4.0 mg/kg/day for artemisinin derivatives. The higher maximum dose of artemether (8.0 mg/kg/day) is not expected to result in any drug‐related adverse events and has been demonstrated to be safe when administered both once daily and twice daily as oral artesunate for 3 days in patients with uncomplicated *P. falciparum* malaria.^20^ The higher dosing of lumefantrine (**Table** **S1**) does not translate into a substantially increased drug exposure due to the dose‐limited absorption of lumefantrine, resulting in less than proportional increase in the absorption of lumefantrine with increasing dosing.^13^ Increased dosing of lumefantrine should therefore have a minimal impact on efficacy or risk for adverse events, but a higher dose might reduce acute GI tolerability. The overall daily dose of amodiaquine in the proposed TACT regimen (6–16 mg/kg/day, **Table** **S3**) is comparable to the currently recommended dose when administered as artesunate‐amodiaquine (6.75–15 mg/kg/day, **Table** **S2**). However, this daily dose is split into two dose occasions for the TACT, resulting in only half of the daily dose being administered every 12 hours. This leads to substantially reduced peak concentrations while maintaining the systemic exposure to amodiaquine and its active metabolite, desethylamodiaquine. There have been tolerability issues reported for all amodiaquine‐containing regimens currently on the market, which are associated with higher mg/kg dosing of amodiaquine.^21^ These tolerability issues are acute GI effects and, therefore, associated with poor local GI tolerability of high doses or with high peak concentrations, rather than with high total systemic exposures. Thus, this novel fixed‐dose co‐formulated TACT might be better tolerated than currently available ACTs.

There are several limitations to this study. The published pharmacokinetic models have been developed for the currently available ACTs and we extrapolate these by assuming that the pharmacokinetic properties of each drug will be unaffected by the combination. We believe that this is a fair assumption as no pharmacokinetic or safety‐related drug–drug interactions of clinical concern have been reported in the trials conducted so far. Published pharmacokinetic models were based on current ACT dosing recommendations and we assume in these simulations that models can be extrapolated to the increased doses evaluated here. This has not been explicitly shown, but it is likely that the pharmacokinetic properties within the normal dose range can also be extended to the dosing range evaluated here. A large database of real‐world patients was used to estimate the overall relationship between age and body weight for simulations. This relationship was used to generate virtual patients with biologically plausible body weight and age combinations. Thus, virtual patients were not bootstrapped from the database and the expected variability will, therefore, be reduced as patients with skewed age‐weight relationships were not represented in the simulations. However, the aim of this work was to develop an optimal dosing by achieving an equivalent mean exposure in children compared to adults. Additional variability between patients will not impact the results and conclusions from this dose optimization approach.

In conclusion, we propose a fixed‐dose formulation of artemether‐lumefantrine‐amodiaquine (20:120:40 mg) to simplify manufacturing, implementation, and further development of a co‐formulated product. Three published population‐based pharmacometric models and two large cohorts of observed adult and pediatric malaria patients were used to simulate pharmacokinetic profiles of different dosing strategies. Based on total exposure and peak concentrations, an optimal dose regimen was developed resulting in an extension of the current 4 weight bands to a total of 5 weight bands to generate equivalent exposures in all body weight groups and minimize the fluctuation in exposure between patients. This optimal dosing maximizes the chance of patient cure by providing an increased artemether‐lumefantrine mg/kg dose in small children and large adults while minimizing the risk of increased adverse events linked to higher doses of amodiaquine by splitting the standard once‐daily dose into twice daily dosing to be co‐formulated with artemether‐lumefantrine.

## FUNDING

This work was partly supported by the Wellcome Trust (220211). For the purpose of open access, the author has applied a CC BY public copyright license to any Author Accepted Manuscript version arising from this submission.

## CONFLICT OF INTEREST

The authors declared no competing interests for this work.

## AUTHORS CONTRIBUTIONS

J.T., N.J.W., and A.M.D. wrote the manuscript; J.T., N.J.W., and A.M.D. designed the research; J.T. performed the research; and J.T. analyzed the data.

## Supporting information


Data S1

## Data Availability

All modeling and simulation codes are published and freely available from the open access publications. For any other information, contact the first author.
