# Supplementary material for: Dose‐Optimization of a Novel Co‐Formulated Triple Combination Antimalarial Therapy: Artemether‐Lumefantrine‐Amodiaquine
Source: Clin Pharmacol Ther. 2025 Feb 12;117(5):1248–53. doi: 10.1002/cpt.3582 (PMC11993281; doi:10.1002/cpt.3582)
Supplement: Supplementary file 1 — Data S1 [file CPT-117-1248-s001.pdf]

Supplementary information

Dose-Optimization of a Novel Co-Formulated Triple Combination Antimalarial Therapy: Artemether-Lumefantrine-Amodiaquine

Joel Tarning, Nicholas J. White, Arjen M. Dondorp

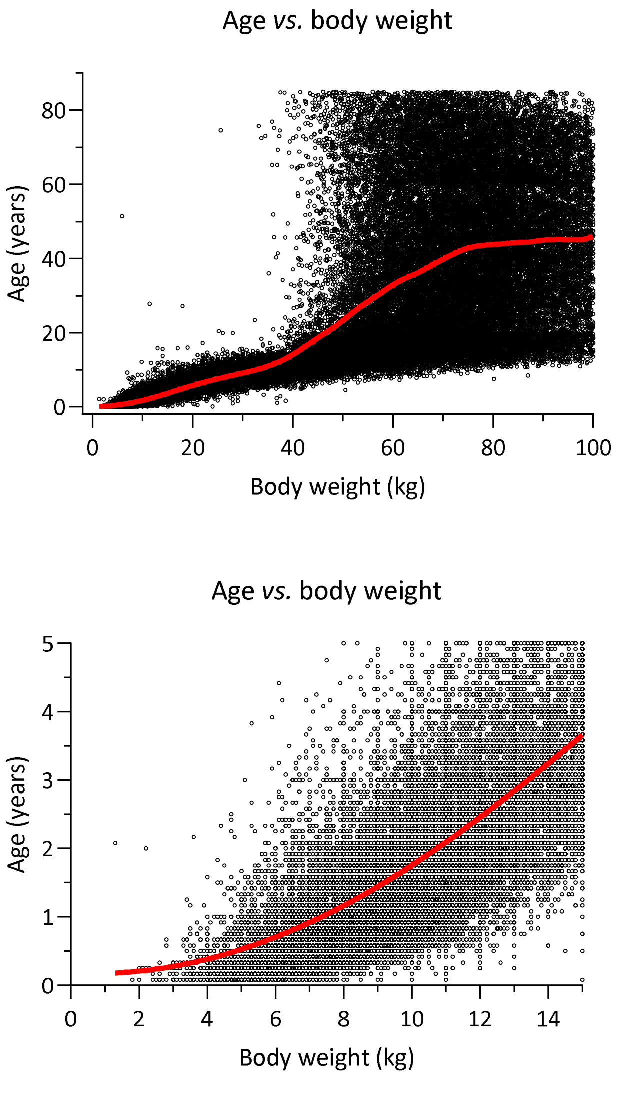

Figure S1. Age *vs.* body weight demographics in adult and paediatric patients.

Open circles are patient demographics obtained from two publicly available data bases; the National Health and Nutritional Examination Survey (NHANES III) from the US CDC (n = 53,833) and the Severe Malaria African Children (SMAC) network (n = 26,051). The red solid line is a locally-weighted scatterplot smoothing (10-point smoothing window), applied to all age-for-weight data, using GraphPad Prism v.10.2.2.

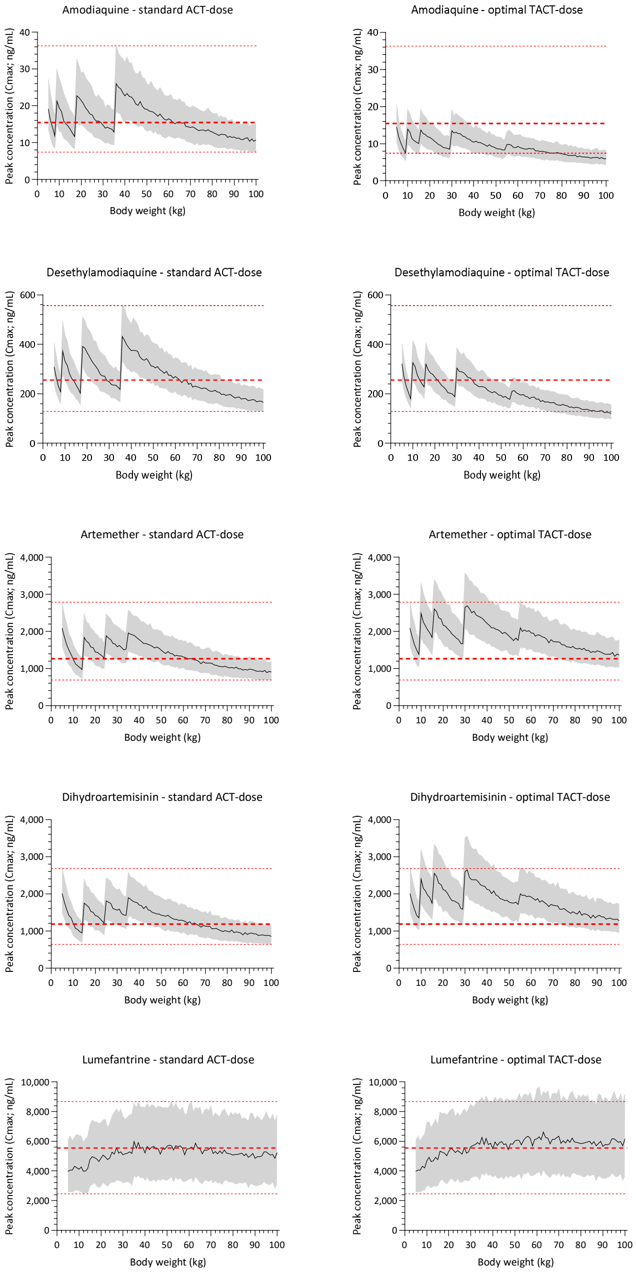

Figure S2. Simulated peak concentration of amodiaquine/desethylamodiaquine, artemether/dihydroartemisinin, and lumefantrine after standard ACT-dosing and proposed novel TACT-dosing.

Peak concentration is derived as the maximum plasma concentration in the concentration-time profile (C_{MAX}) for each simulated individual. Each dosing simulation is based on 96,000 virtual patients, distributed uniformly between 5 and 100 kg body weight, and shown as median (solid line) and interquartile range (grey shaded area) of simulated peak concentrations. Dashed red lines are the median peak concentrations in a typical patient at 60 kg, receiving standard dosing. Dotted red lines are simulated minimum and maximum interquartile peak concentrations associated with standard dosing.

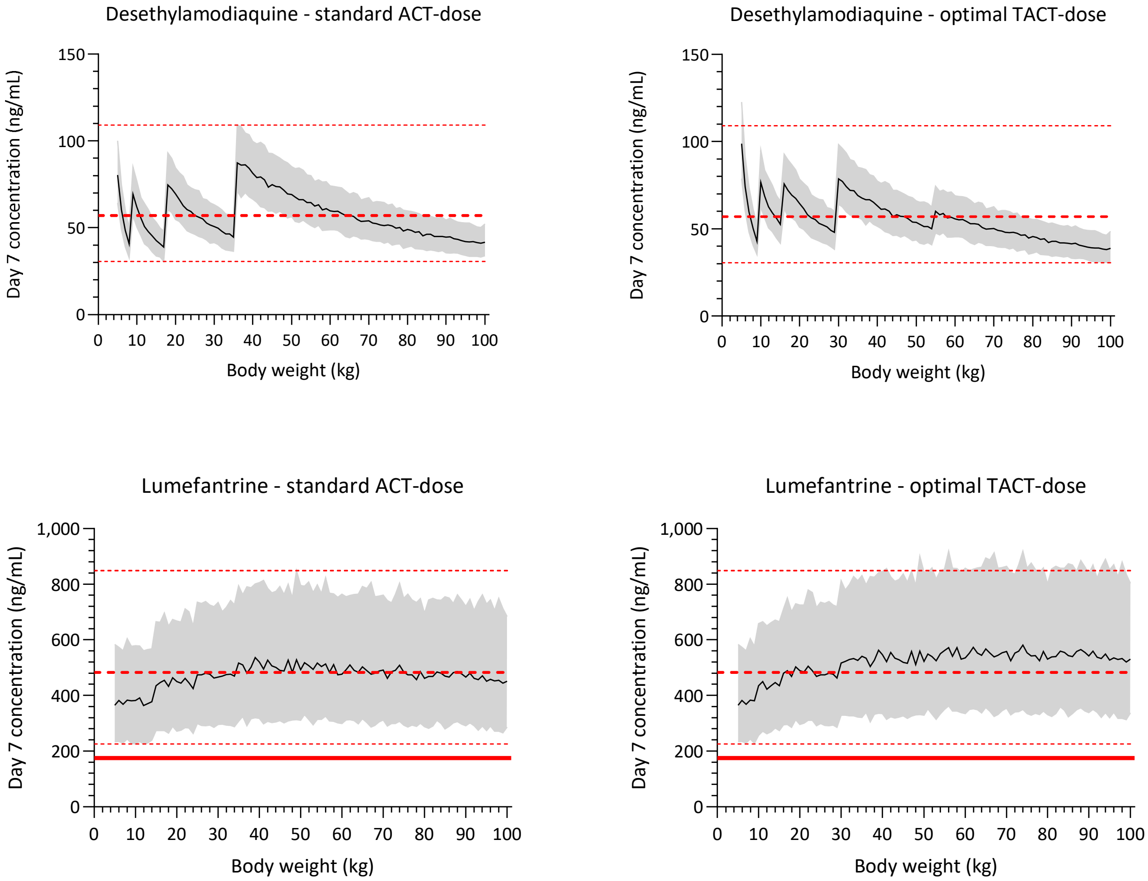

Figure S3. Simulated day 7 concentration of amodiaquine/desethylamodiaquine and lumefantrine after standard ACT-dosing and proposed novel TACT-dosing.

Day 7 concentration is derived from the plasma concentration-time profile for each simulated individual. Each dosing simulation is based on 96,000 virtual patients, distributed uniformly between 5 and 100 kg body weight, and shown as median (solid line) and interquartile range (grey shaded area) of simulated peak concentrations. Dashed red lines are the median peak concentrations in a typical patient at 60 kg, receiving standard dosing. Dotted red lines are simulated minimum and maximum interquartile peak concentrations associated with standard dosing. The thick solid red line at 175 ng/mL of lumefantrine is a previously published threshold associated with therapeutic success.

Table S1. Current dosing of artemether-lumefantrine; twice daily dosing

Body weight (kg)	Artemether (mg/day)	Artemether (mg/kg/day)	Lumefantrine (mg/day)	Lumefantrine (mg/kg/day)
5 to 14	40	2.52 – 4.00	240	15.09 – 24.00
15 to 24	80	2.68 – 5.00	480	16.05 – 30.00
25 to 34	120	2.19 – 4.00	720	13.11 – 24.00
35 to 80	160	2.00 – 2.91	960	12.00 – 17.45

Table S2. Current dosing of artesunate-amodiaquine; once daily dosing

Body weight (kg)	Artesunate (mg/day)	Artesunate (mg/kg/day)	Amodiaquine (mg/day)	Amodiaquine (mg/kg/day)
5 to 8	25	3.13 – 5.00	67.5	8.45 - 13.50
9 to 17	50	2.94 - 5.56	135	7.94 – 15.00
18 to 35	100	2.86 - 5.56	270	7.71 – 15.00
36 to 80	200	2.50 - 5.56	540	6.75 – 15.00

Table S3. Proposed optimal dosing of a novel fixed-dose combination of artemether-lumefantrine-amodiaquine; twice daily dosing

Body weight (kg)	Artemether (mg/day)	Artemether (mg/kg/day)	Lumefantrine (mg/day)	Lumefantrine (mg/kg/day)	Amodiaquine (mg/day)	Amodiaquine (mg/kg/day)
5 to 9.9	40	4.04 – 8.00	240	24.242 – 48.00	80	8.08 – 16.00
10 to 15.9	80	5.03 – 8.00	480	30.19 – 48.00	160	10.06 – 16.00
16 to 29.9	120	4.01 – 7.50	720	24.08 – 45.00	240	8.03 – 15.00
30 to 54.9	200	3.64 – 6.67	1200	21.86 – 40.00	400	7.29 – 13.33
55 to 80	240	3.00 – 4.36	1440	18.00 – 26.18	480	6.00 – 8.73

Appendix 1 - NONMEM code for Artemether and Dihydroartemisinin

```
;;-------------------------------------------------------------------------------;;
;;  NONMEM model template created by MORU
;;  Modeller: Prof. Joel Tarning
;;  Reference: Onyamboko et al., AAC, 2020
;;  Simulate artemether (ARM) and dihydroartemisinin (DHA)
;;  Date: 2024-12-01
;;-------------------------------------------------------------------------------;;

$PROBLEM 1

$INPUT
 ID TIME AMT DV MDV EVID CMT WT EGA

$DATA
 simulation_data.csv                    ; Data input file

$SUBROUTINE
 ADVAN13 TOL = 6

$MODEL
 COMP = (DOS)                           ; 1 (Absorption, Dose)
 COMP = (ARM)                           ; 2 (Central, ARM)
 COMP = (DHA)                           ; 3 (Central, DHA)
 COMP = (TRAN1)                         ; 4 (Transit 1)
 COMP = (TRAN2)                         ; 5 (Transit 2)
 COMP = (TRAN3)                         ; 6 (Transit 3)
 COMP = (TRAN4)                         ; 7 (Transit 4)
 COMP = (TRAN5)                         ; 8 (Transit 5)
 COMP = (TRAN6)                         ; 9 (Transit 6)

$PK
;;--- F1EGA-DEFINITION START -----------------------------------------------------;;
 F1EGA = (1 + THETA(7) * (EGA - 48.5))   ; Gestational age-dependent bioavailability
;;--- F1EGA-DEFINITION END -------------------------------------------------------;;
```

```
;;--- Artemether (ARM) -------------------------------------------------------------;;

 TVF1  = THETA(1) * F1EGA;
 F1    = TVF1 * EXP(ETA(1));

 TVMT  = THETA(2);
 MT    = TVMT * EXP(ETA(2));

 TVCL  = THETA(3) * ((WT/53)**0.75);
 CL    = TVCL * EXP(ETA(3));

 TVV2  = THETA(4) * ((WT/53)**1);
 V2    = TVV2 * EXP(ETA(4));

;;--- Dihydroartemisinin (DHA) -------------------------------------------------------;;

 TVCLM = THETA(5) * ((WT/53)**0.75);
 CLM   = TVCLM * EXP(ETA(5));

 TVV3  = THETA(6) * ((WT/53)**1);
 V3    = TVV3 * EXP(ETA(6));

 S2 = V2 / 1000                        ; Scaling, dose (mg) -> conc. (ng/mL)
 S3 = V3 / 1000                        ; Scaling, dose (mg) -> conc. (ng/mL)

 MW_ARM = 298.37                       ; Molecular weight ARM (g/mol)
 MW_DHA = 284.35                       ; Molecular weight DHA (g/mol)
 CF     = MW_DHA / MW_ARM              ; Conversion factor ARM -> DHA

 nn  = 6
 KTR = (nn + 1) / MT

 K14 = KTR
 K45 = KTR
```

```
 K56 = KTR
 K67 = KTR
 K78 = KTR
 K89 = KTR
 K92 = KTR

 K23 = CL / V2
 K30 = CLM / V3

$DES
 DADT(1) = - KTR*A(1)
 DADT(4) = KTR*A(1) - KTR*A(4)
 DADT(5) = KTR*A(4) - KTR*A(5)
 DADT(6) = KTR*A(5) - KTR*A(6)
 DADT(7) = KTR*A(6) - KTR*A(7)
 DADT(8) = KTR*A(7) - KTR*A(8)
 DADT(9) = KTR*A(8) - KTR*A(9)

 DADT(2) = KTR*A(9) - K23*A(2)

 DADT(3) = CF*K23*A(2) - K30*A(3)

$ERROR
 IF (CMT.EQ.2) CP = A(2) / S2            ; Venous concentration ARM (ng/mL)
 IF (CMT.EQ.3) CP = A(3) / S3            ; Venous concentration DHA (ng/mL)
 IPRED = CP
 IF (CMT.EQ.2) Y  = IPRED + IPRED * EPS(1)
 IF (CMT.EQ.3) Y  = IPRED + IPRED * EPS(2)

$THETA
 1.0                                     ; 1.F1
 0.487                                   ; 2.MTT
 365                                     ; 3.CL/F ARM
 1350                                    ; 4.Vc/F ARM
 363                                     ; 5.CL/F DHA
```

```
 110                                    ; 6.Vc/F DHA
 -0.00175                               ; 7.F1-EGA

$OMEGA
 0.12507                                ; 1.IIV F1 ARM
 0.37358                                ; 2.IIV MTT ARM
 0.04117                                ; 3.IIV CL/F ARM
 0.03017                                ; 4.IIV Vc/F ARM
 0.06348                                ; 5.IIV CL/F DHA
 0 FIX                                  ; 6.IIV Vc/F DHA

$SIGMA
 0.549                                  ; RUV ARM
 0.474                                  ; RUV DHA

$SIM
 (546345) (52921) ONLYSIM SUBPROBLEMS=1000
```

Appendix 2 – NONMEM code for Lumefantrine

```
;;----------------------------------------------------------------------------------;;
;; NONMEM model template created by MORU
;; Modeller: Prof. Joel Tarning
;; Reference: Kloprogge et al., PLOS Med, 2018
;; Simulate Lumefantrine (LF)
;; Date: 2024-12-01
;;----------------------------------------------------------------------------------;;

$PROBLEM 2

$INPUT
 ID TIME AMT DV MDV EVID CMT WT PBM MGKG PREG

$DATA
 simulation_data.csv                      ; Data input file

$SUBROUTINE
 ADVAN13 TOL = 6

$MODEL
 COMP = (DOS)                             ; 1 (Absorption, Dose)
 COMP = (VC_LF)                           ; 2 (Central, LF)
 COMP = (VP_LF)                           ; 3 (Peripheral, LF)

$PK
;;--- F1PBM-DEFINITION START --------------------------------------------------------;;
 F1PBM = ((PBM / 4.20)**THETA(10))        ; Parasite density-dependent bioavailability
;;--- F1PBM-DEFINITION END ----------------------------------------------------------;;

;;--- F1DOSE-DEFINITION START -------------------------------------------------------;;
 F1DOSE = 1 - (MGKG / (THETA(9) + MGKG))  ; Dose-dependent bioavailability
;;--- F1DOSE-DEFINITION END ---------------------------------------------------------;;
```

```
;;--- F1-RELATION START-------------------------------------------------------------;;
 F1COV = F1PBM * F1DOSE
;;--- F1-RELATION END --------------------------------------------------------------;;

;;--- KAPREG-DEFINITION START ------------------------------------------------------;;
 IF(PREG.EQ.0) KAPREG = 1                 ; Pregnant absorption rate
 IF(PREG.EQ.1) KAPREG = ( 1 + THETA(8))   ; Non-pregnant absorption rate
;;--- KAPREG-DEFINITION END --------------------------------------------------------;;

 TVCL  = THETA(1) * ((WT/42)**0.75);
 CL    = TVCL * EXP(ETA(1));

 TVV2  = THETA(2) * ((WT/42)**1);
 V2    = TVV2 * EXP(ETA(2));

 TVQ   = THETA(3) * (WT/42)**0.75
 Q     = TVQ * EXP(ETA(3))

 TVV3  = THETA(4) * (WT/42)**1
 V3    = TVV3 * EXP(ETA(4))

 TVKA  = THETA(5) * KAPREG
 KA    = TVKA * EXP(ETA(5))

 TVF1  = THETA(6) * F1COV
 BXPAR = THETA(7)
 PHI   = EXP(ETA(6))
 ETATR = (PHI**BXPAR - 1) / BXPAR
 F1    = TVF1 * EXP(ETATR)

 S2 = V2 / 1000                          ; Scaling, dose (mg) -> conc. (ng/mL)

 K12 = KA
 K23 = Q / V2
 K32 = Q / V3
```

```
 K20 = CL / V2

$ERROR
 CP    = A(2) / S2                          ; Venous concentration LF (ng/mL)
 IPRED = CP
 Y     = IPRED + IPRED * EPS(1)

$THETA
 1.35                                       ; 1.CL/F
 11.2                                       ; 2.Vc/F
 0.344                                      ; 3.Q/F
 59.0                                       ; 4.Vp/F
 0.0386                                     ; 5.KA
 1.0                                        ; 6.F1
 -0.343                                     ; 7.Boxcox shape parameter
 0.352                                      ; 8.KA-Pregnancy
 3.86                                       ; 9.F1-Dose50 (mg/kg)
 -0.643                                     ; 10.F1-Parasitemia

$OMEGA
 0 FIX                                      ; 1.IIV CL/F
 1.120                                      ; 2.IIV Vc/F
 0 FIX                                      ; 3.IIV Q/F
 0 FIX                                      ; 4.IIV Vp/F
 0 FIX                                      ; 5.IIV KA
 0.402                                      ; 6.IIV F1

$SIGMA
 0.323                                      ; RUV

$SIM
 (546345) (52921) ONLYSIM SUBPROBLEMS=1000
```

Appendix 3 – NONMEM code for Amodiaquine and Desethylamodiaquine

```
;;-------------------------------------------------------------------------------------;;
;;  NONMEM model template created by MORU
;;  Modeller: Prof. Joel Tarning
;;  Reference: Ali et al., AAC, 2018
;;  Simulate amodiaquine (AQ) and desethylamodiaquine (DEAQ)
;;  Date: 2024-12-01
;;-------------------------------------------------------------------------------------;;

$PROBLEM 3

$INPUT
 ID TIME AMT DV MDV EVID CMT OCC WT PMA

$DATA
 simulation_data.csv                    ; Data input file

$SUBROUTINE
 ADVAN13 TOL = 6

$MODEL
 COMP = (DOS)                           ; 1 (ABSORB,DOSE)
 COMP = (VC_AQ)                         ; 2 (Central, AQ)
 COMP = (VP_AQ)                         ; 3 (Peripheral, AQ)
 COMP = (VC_DEAQ)                       ; 4 (Central, DEAQ)
 COMP = (VP1_DEAQ)                      ; 5 (Peripheral 1, DEAQ)
 COMP = (VP2_DEAQ)                      ; 6 (Peripheral 2, DEAQ)
 COMP = (TRAN1)                         ; 7 (Transit 1)
 COMP = (TRAN2)                         ; 8 (Transit 2)

$PK
;;--- F1OCC-DEFINITION START ---------------------------------------------------------;;
 TCOV = 0
 IF(OCC.EQ.1) TCOV = 1
 F1_OCC = (1 - THETA(14) * TCOV)        ; Bioavailability for the first dose occasion
```

```
;;--- F1OCC-DEFINITION END ---------------------------------------------------------;;

;;--- CLAGE-DEFINITION START -------------------------------------------------------;;
 PMA = (AGE * 12) + 9                    ; Postmenstrual age + postnatal age (months)
 CL_AGE_AQ = PMA**THETA(16) / (PMA**THETA(16) + THETA(15)**THETA(16))
 CL_AGE_DEAQ = PMA**THETA(18) / (PMA**THETA(18) + THETA(17)**THETA(18))
;;--- CLAGE-DEFINITION END ---------------------------------------------------------;;

;;--- Amodiaquine (AQ) --------------------------------------------------------------;;

 TVF1  = THETA(1) * F1_OCC
 F1    = TVF1*EXP(ETA(1))

 TVMT  = THETA(2)
 MT    = TVMT * EXP(ETA(2))

 TVKA  = THETA(3)
 KA    = TVKA * EXP(ETA(3))

 TVCL  = THETA(4) * ((WT/50)**0.75) * CL_AGE_AQ
 CL    = TVCL * EXP(ETA(4))

 TVV2  = THETA(5) * (WT/50)**1
 V2    = TVV2 * EXP(ETA(5))

 TVQ   = THETA(6) * (WT/50)**0.75
 Q     = TVQ * EXP(ETA(6))

 TVV3  = THETA(7) * (WT/50)**1
 V3    = TVV3 * EXP(ETA(7))

;;--- Desethylamodiaquine (DEAQ) ----------------------------------------------------;;

 TVCLM = THETA(8) * ((WT/50)**0.75) * CL_AGE_DEAQ
 CLM   = TVCLM * EXP(ETA(8))
```

```
TVV4  = THETA(9) * (WT/50)**1
V4    = TVV4 * EXP(ETA(9))

TVQ2  = THETA(10) * (WT/50)**0.75
Q2    = TVQ2 * EXP(ETA(10))

TVV5  = THETA(11) * (WT/50)**1
V5    = TVV5 * EXP(ETA(11))

TVQ3  = THETA(12) * (WT/50)**0.75
Q3    = TVQ3 * EXP(ETA(12))

TVV6  = THETA(13) * (WT/50)**1
V6    = TVV6 * EXP(ETA(13))

S2 = V2 / 1000                          ; Scaling, dose (mg) -> conc. (ng/mL)
S4 = V4 / 1000                          ; Scaling, dose (mg) -> conc. (ng/mL)

MW_AQ   =  355.87                       ; Molecular weight AQ (g/mol)
MW_DEAQ =  327.813                      ; Molecular weight DEAQ (g/mol)
CF      = MW_DEAQ / MW_AQ               ; Conversion factor AQ -> DEAQ

nn  = 2
KTR = (nn + 1) / MT

K17 = KTR
K78 = KTR
K82 = KA

K23   = Q/V2
K32   = Q/V3
K24   = CL/V2

K45   = Q2/V4
```

```
 K54   = Q2/V5
 K46   = Q3/V4
 K64   = Q3/V6
 K40   = CLM/V4

$DES
 DADT(1) = - KTR*A(1)
 DADT(7) = KTR*A(1) - KTR*A(7)
 DADT(8) = KTR*A(7) - KA*A(8)

 DADT(2) = KA*A(8) - K23*A(2) + K32*A(3) - K24*A(2)
 DADT(3) = K23*A(2) - K32*A(3)

 DADT(4) = CF*K24*A(2) - K45*A(4) + K54*A(5) - K46*A(4) + K64*A(6) - K40*A(4)
 DADT(5) = K45*A(4) - K54*A(5)
 DADT(6) = K46*A(4) - K64*A(6)

$ERROR
 IF (CMT.EQ.2) CP = A(2) / S2            ; Venous concentration AQ (ng/mL)
 IF (CMT.EQ.4) CP = A(4) / S4            ; Venous concentration DEAQ (ng/mL)
 IPRED = CP
 IF (CMT.EQ.2) Y  = IPRED + IPRED*EPS(1) + EPS(2) + 0.2
 IF (CMT.EQ.4) Y  = IPRED + IPRED*EPS(3) + 0.2

$THETA
 1.0                                     ; 1.F1 AQ
 0.236                                   ; 2.MTT AQ
 0.589                                   ; 3.KA AQ
 2960                                    ; 4.CL/F AQ
 13500                                   ; 5.Vc/F AQ
 2310                                    ; 6.Q/F AQ
 22700                                   ; 7.Vp/F AQ
 32.6                                    ; 8.CL/F DEAQ
 258                                     ; 9.Vc/F DEAQ
 154                                     ; 10.Q1/F DEAQ
```

```
 2460                                        ; 11.Vp1/F DEAQ
 31.3                                        ; 12.Q2/F DEAQ
 5580                                        ; 13.Vp2/F DEAQ
 0.224                                       ; 14.F1_OCC
 11.8                                        ; 15.MF50 AQ
 3.6                                         ; 16.Hill AQ
 12.9                                        ; 17.MF50 DEAQ
 3.22                                        ; 18.Hill DEAQ

$OMEGA
 0.095481                                    ; 1.IIV F1 AQ
 0.872356                                    ; 2.IIV MTT AQ
 0.616225                                    ; 3.IIV KA AQ
 0.103684                                    ; 4.IIV CL/F AQ
 0.281961                                    ; 5.IIV Vc/F AQ
 0 FIX                                       ; 6.IIV Q/F AQ
 0 FIX                                       ; 7.IIV Vp/F AQ
 0.004                                       ; 8.IIV CL/F DEAQ
 0.451584                                    ; 9.IIV Vc/F DEAQ
 0 FIX                                       ; 10.IIV Q1/F DEAQ
 0 FIX                                       ; 11.IIV Vp1/F DEAQ
 0 FIX                                       ; 12.IIV Q2/F DEAQ
 0 FIX                                       ; 13.IIV Vp2/F DEAQ

$SIGMA
 0.039601                                    ; Proportional RUV AQ
 0.445                                       ; Additive RUV AQ
 0.058564                                    ; Proportional RUV DEAQ

$SIM
 (546345) (52921) ONLYSIM SUBPROBLEMS=1000
```
